# A Fruitful Decade Using Synthetic Promoters in the Improvement of Transgenic Plants

**DOI:** 10.3389/fpls.2019.01433

**Published:** 2019-11-01

**Authors:** Sajid Ali, Won-Chan Kim

**Affiliations:** School of Applied Biosciences, Kyungpook National University, Daegu, South Korea

**Keywords:** synthetic promoters, transgenes, plant improvement, gene expression, drought

## Abstract

Advances in plant biotechnology provide various means to improve crop productivity and greatly contributing to sustainable agriculture. A significant advance in plant biotechnology has been the availability of novel synthetic promoters for precise spatial and temporal control of transgene expression. In this article, we review the development of various synthetic promotors and the rise of their use over the last several decades for regulating the transcription of various transgenes. Similarly, we provided a brief description of the structure and scope of synthetic promoters and the engineering of their *cis*-regulatory elements for different targets. Moreover, the functional characteristics of different synthetic promoters, their modes of regulating the expression of candidate genes in response to different conditions, and the resulting plant trait improvements reported in the past decade are discussed.

## Introduction

The application of plant biotechnology techniques has revolutionized the field of plant science and greatly contributed to the progress of crop improvement and modern agriculture (Moose and Mumm 2008; Hahne et al., 2011). The demands of an expanding global population and an expectation of higher living standards are two main forces driving the widespread implementation of innovative agro-biotechnology concepts and techniques. Many crops have been genetically modified for high yield, stress tolerance, and production of diverse high-value products, such as antibodies, enzymes, vaccines, and other bioactive secondary metabolites (Goodman et al., 1987; Gasser and Fraley 1989; Saito et al., 1992; De Jaeger et al., 2002; Rushton et al., 2002; Gust et al., 2010; Madanala et al., 2015; Kashima et al., 2016). Plant biotechnology has been successfully used to improve crops for greater productivity under both normal and stressful conditions (Sakamoto and Murata 2002; Wang et al., 2003; Nessler et al., 2015). During the past three decades, the progress and application of molecular biology, plant biotechnology, and synthetic biology have generated new tools for the design and analysis of genetically modified organisms (Sharma et al., 2002; Moose and Mumm 2008; Nessler et al., 2015).

The process of transcription is an essential cell function for gene regulation which is accomplished through sequence-specific binding transcription factors binding to their target promoters and either inhibiting or activating transcription. The pivotal role in controlling processes is not played by encoding sequences, but by regulatory elements (Venter and Botha 2010). Where, regulatory sequences dynamically enhance or restrict gene expression levels within an organism. Hence, controlled expression of genetic information leads to the production of the required amount of the final product. Similarly, regulatory sequences perform an indispensable role in regulation and promoter regulate the expression of a transgene in different ways to achieve inducible or constitutive expression. In addition, other elements of the host genome participate in controlling the transgene’s expression through interactions with DNA or endogenously expressed regulatory proteins at different stages. The structure of plant promoters allows to construct various sets of regulatory elements and plant promoters for genes transcribed by RNA polymerase II have been studied extensively (Guilfoyle 1997). The understanding of regulation of plant gene expression at *cis*-acting elements started with analysis of promoters from native plant genes and *Agrobacterium* which express in plant cells. Interestingly, like their counterparts in animals, plant gene promoters contain TATA box, initiator element as well as well-defined transcription initiation site about 20 to 30 bp downstream to TATA element. Whereas, in the upstream region, several gene promoters were found to contain positive or negative regulatory elements, some of which were characterized as enhancers or silencers (Timko et al., 1985; Tyagi 2001). A minimal promoter (mostly –46 bp derivative of 35S *CaMV* promoter or its –90 bp derivative) is fused to a heterologous promoter sequence at its 5´ end and to a reporter gene (GUS, LUC, CAT, etc) at its 3´ end. Such constructs are introduced in plant cells by *Agrobacterium*, electroporation, or biolistics and evaluated either for transient expression of the reporter gene or for its expression in stable transgenic system. The pattern of expression conferred by heterologous promoter sequences has been helpful in defining characteristics of developmental as well as inducible regulatory promoters (Tyagi et al., 1999; Tyagi 2001).

Tightly controlled promoters can achieve very specific expression patterns for transgenes, and studies have elucidated many aspects of the underlying cellular regulation mechanisms by using synthetic promoters, in which primary elements of different promoters from diverse origins are linked together. Primary elements of a synthetic promoter is the region known as a regulatory module that is fused upstream to the core promoter (Venter 2007; Dey et al., 2015; Kassaw et al., 2018). Precisely, a synthetic promoter is a stretch of DNA comprising a core-promoter region and multiple repeats or combinations of heterologous upstream regulatory elements (*cis*-motifs or TF-binding sites). The core-promoter region (also known as the minimal-region) usually contains a TATA-box necessary for recruiting RNA polymerase II and the assembly of general transcription factors to form the preinitiation complex. Thus, the multiplication of the elements, their reorganization, and ligation with native and synthetic *cis*-sequences are used to obtain synthetic promoters. The development and use of such a novel promoter can successfully regulate transgenes in trait- and environment-specific manners (Venter and Botha 2010; Dey et al., 2015; Biłas et al., 2016; Mohan et al., 2017).

The basic idea behind synthetic promoter is to improve the expression characteristics so as to make promoters that are more suited to the biotechnological aim. The common goals of synthetic promoters are to reduce unwanted background expressions and increase promoter strength. Typically, synthetic promoters are constructed by using *Cis*-acting element building blocks from various sources. The combinatorial engineering of *cis*-regulatory elements include activators, enhancers, and repressors, in the region upstream of core promoter sequences. The different *cis*-acting elements in plant promoters can be substituted or shuffled in order to achieve a desired regulatory function (Mohan et al., 2017; Kassaw et al., 2018). Different factors such as orientation, location, and copy number also affect the functional role of synthetically designed regulatory elements (Venter 2007; Dey et al., 2015). *Cis*-regulatory elements that are placed upstream or downstream of a native promoter can be altered to achieve desired effects. The transcription factors that attach strongly to *cis*-regulatory elements in synthetic promoters can not only enhance the process of transcription but also influence other regulatory effects on the target gene. Categorically, investigations of different aspects, such as specificity, spacing, and copy number, of *cis*-regulatory elements and their corresponding transcription factors have demonstrated that synthetic promoters can be more efficient compared to native or natural promoters. The tight control of synthetic promoters is supported by the interactions among *cis*-motifs and their corresponding transcription factors for enhanced transcription (Mehrotra et al., 2011; Dey et al., 2015). The researchers in plant synthetic promoter engineering are advancing to generate robust constitutive, inducible or bidirectional synthetic promoters for a better transcriptional regulation of transgene expression in model and crop plants. *Several synthetic promoters have been tested for fusion of specific Cis*-regulatory elements with a core promoter or hybrids of multiple promoter, parts from different regulatory sequences. Similarly, various synthetic promoters have been designed by inserting functional promoter elements into natural plant promoters (**Tables 1**–**3**).

**Table 1:** Demonstrates constitutive synthetic promoters (CSPs) and their characteristics.

Name/ID/Title	Source	Expression	Species tested	Reference
12–10, 12–48, 12–79	*Ubiquitin1* transcripts	Constitutive expression	*Physcomitrella patens*	Peramuna et al., 2018
AZprom-1 to AZprom-21	CaMV 35S and Ribulose-1,5-bisphosphate carboxylase small subunit promoter	Constitutive expression	*Nicotiana tabacum* var Samsun	Venter and Zwiegelaar 2017 (Patent)
*Saps (Sap11)*	POWRS motifs, GC content, AT-rich and TC rich motifs	Constitutive expression	*Chlamydomonas reinhardtii*	Scranton et al., 2016
TGA1(CmYLCV)	Cis-elements of Cestrum Yellow Leaf Curling Virus (CmYLCV) distal promoter region	Constitutive expression	*Nicotiana tabacum*, *Arabidopsis*	Sahoo et al., 2016
SynS1, SynS2	Different Cis-elements in the synthetic module (SynS)	Constitutive expression	Saccharum officinarum	Chakravarthi et al., 2015
MUASMSCP	Upstream activation sequence of *Mirabilis mosaic virus*, promoter-fragment along with TATA element.	Constitutive expression	*Nicotiana tabacum* and *Arabidopsis* protoplasts whole plant of *Petunia hybrid*	Acharya et al., 2014a
MSgt-PFlt, FSgt-PFlt, PFlt-UAS-2X	Sub-genomic transcript promoter from *Figwort Mosaic Virus*	Constitutive expression	*Nicotiana tabacum*, Spinach, *Arabidopsis, Petunia hybrida, and Solanum lycopersicum*	Acharya et al., 2014b
FfC (FUAS35SCP), FsFfCBD (FS5FUAS35SCPBD)	FMVFlt, MMVFlt, and CaMV35S	Bidirectional constitutive expression	*Nicotiana tabacum*	Patro et al., 2013
FSuasFcp, FuasFScp	FMVSgt and FMVFlt	Constitutive expression	*Nicotiana tabacum*	Ranjan and Dey, 2012
MSgt-FSgt	UAS of MMVSgt and Core domain of FMVSgt	Constitutive expression	Transgenic *Nicotiana tabacum* and *Arabidopsis*	Kumar et al., 2011
VR-ACS1	Vigna ACC synthase (*aminocyclopropane-1- carboxylate synthase*) gene promoter	Constitutive expression	*Vigna radiate*	Wever et al., 2010
pOCSn-OCS, pLOCSn-OCS, pΔOCS, pLOCSΔOCS	*Ocs* element of Octopine synthase promoter	Constitutive expression	Transgenic *Nicotiana tabacum*	Liu and Bao 2009

**Table 2:** Demonstrates different types of inducible synthetic promoters (ISPs) and their characteristics.

Name/ID	Source	Expression	Species tested	Reference
Ap, Dp, ANDp	Synthetic promoters were designed based on the promoters of RD29A and RD29B, DRE (A/GCCGAC), as a cis-element, The sequences of RD29A and RD29B promoters	Inducible expression	*Arabidopsis thaliana*	Ge et al., 2018
*P_DRE::35S*	Core element of CaMV*35S* promoter, TMV omega 5′-UTR, *35S* core sequence, cold	Inducible expression	*Arabidopsis*	Gerasymenko and Sheludko 2017
SINC, GmubiSINC	5′ UTR of soybean polyubiquitin promoter, Upstream CaMV 35S	Inducible expression	*Glycine max*	Grant et al., 2017
SP-DDEE	Parsley *D, E17*elements and minimal promoter	Inducible expression	*Brassica napus*	Moradyar et al., 2016
GWH	Cis-element include SARE, JERE, GCC 2× HSRE, and 6× W-box	Inducible expression	*Arabidopsis*	Zhu et al., 2015
*p4xKST82-rd29B*	Potato KST1 promoter (4xKST82), and dehydration responsive (rd29B) promoter from Arabidopsis	Inducible expression	*Nicotiana tabacum*	Na and Metzger 2014
*W2X,* *GCC2X,GCC3X,S2X*	−46 region minimal promoter of CaMV 35S, 2 or 3 copies of W, GCC and S boxes in transgenic tobacco plants	Inducible expression	*Nicotiana tabacum*	Yeri et al., 2013
EKCM, EKCRM, ECCRM	Different cis-acting stress response elements were derived from stress induced promoters such as rd29A, erd1, cor15a, kin1 of Arabidopsis	Inducible expression	*Arabidopsis thaliana*	Hou et al., 2012
EFCFS-HS-1, EFCFS-HS-2, EFCFS-HS-3	Created by combining the distal region (−227 to −54, FUAS) of *Figwort mosaic virus* full-length transcript promoter (F20) and the core promoter (FS3CP) domain of *Figwort mosaic virus* sub-genomic transcript promoter (FS3), and also containing ′AAAG′ *cis*-motif (Dof-1)	Inducible expression	*Nicotiana tabacum*	Ranjan and Dey 2012
(SP), SP-EE, SP-FF and SP-FFEE	Pathogen-inducible *cis*-acting elements F and E17, upstream region of *CaMV* 35S minimal promoter	Inducible expression	*Brassica napus*	Shokouhifar et al., 2011
*pporRFP*	Tetramers of certain regulatory elements (4 × RE) were placed upstream of CaMV35S minimal promoter, Enhanced synthetic promoter construct of 4 × RE were placed between B (−415 −90) and A1 (−90 −46) domains of CaMV35S promoter	Inducible expression	*Nicotiana tabacum* cv. Xanthi	Liu et al., 2011
ACGT,(ACGT)_2_,(ACGT)_N5_ (ACGT), (ACGT)_N10_(ACGT), (ACGT)_N25_(ACGT),	Activator ACGT motif (Single or double copies), Pmec minimal promoter	Inducible expression	Arabidopsis and *Nicotiana tabacum* leaves	Mehrotra and Mehrotra 2010
Bs3E/Bs3, Xa27/Bs3,Bs3-E/XA27/Bs3	UPT_AvrXa27_, UPT_AvrBs3rep16_, and UPT_AvrBs3_ Boxes	Inducible expression	*Capsicum annuum*	Römer et al., 2009
2 X W2/2 X S/2 X D, 4 X W2/4 X S	*Cis*-acting elements containing boxes W1, W2, GCC, JERE, S, Gst1, and D. Whereas, each element was inserted between the SpeI and XbaI restriction sites upstream of the CaMv 35S minimal promoter	Inducible expression	*Arabidopsis*	Rushton et al., 2002

**Table 3:** Demonstrate different types of tissue specific synthetic promoters (TSPs) and their characteristics.

Name/ID	Source	Expression	Species tested	Reference
P_RSGA_, P_2RSGA_, P_2RSPA_, P_RSGPA,_ P_2RSGPA_, P_R5SGPA_, P_2R5SGPA_	P_zmBD1_, RY repeats (R), GCN4 (G), Prolamin box (P), Skn-1 (S), ACGT and AACA motifs	Seed specific bidirectional promoters	*Zea mays*	Liu et al., 2018
BiGSSP2, BiGSSP3, BiGSSP6, and BiGSSP7	*P_Osrbcs-550_*, *P_Osrbcs-62_* *OsActin* (*OsAct1*) *OsTubulin6* (*OsTub6I*) already reported sequences	Bidirectional expression efficiencies specifically in green tissues	*Oryza sativa*	Bai et al., 2019
SynR2 SynR1	a. Synthetic module at the 5′ end of the CaMV35S (SynR1), b. module was present in both 5′ and 3′ ends (SynR2)	Root specific	*Nicotiana tabacum*	Mohan et al., 2017
GSSP1, GSSP3, GSSP5, GSSP6, GSSP7	The first intron of rice Act1, G box and GT, Different regulatory sequences from rice, tobacco and Arabidopsis (P_D540-544_, P_Osrbcs-550_, P_Osrbcs-62_, EnP3-110)	Green tissue specific	*Oryza sativa*	Wang et al., 2015
p35S-PCHS -Ω, p35S-LCHS -Ω, pOCSPCHS-Ω, pOCS-LCHS-Ω	Petunia CHSA core promoter, Lily CHS core promoter, Ω element, CaMV 35S or OCS enhancer region	Transgenic enhancement of floral traits (Flower specific)	*Torenia fournieri*	Du et al., 2014
pCL	Created by combining two DNA cassettes: Potato patatin promoter region including a tuber specific sequence TSSR and *Arabidopsis* cor15a promoter region	Express specifically and regulate the activity of acid vacuolar invertase in potato tubers at low temperature	*Solanum tuberosum*	Li et al., 2013
EFCFS-HS-1, EFCFS-HS-2, EFCFSHS-3	EFCFS motif (AAAG),FUAS of F20 along with core promoter of FS3	Tissue specific (Expression of EFCFSHS-3 in Vascular tissues)	*Nicotiana tabacum*	Ranjan and Dey 2012
AtMYB60 promoter::GUS reporters	CaMV35S and AtMYB60 promoters	Tissue specific Expression ells)	*Arabidopsis*	Cominelli et al., 2011
A27znGlb1	Combined the elements of 27zn and Glb1promoters	Tissues specific expressions of the chimeric promoter	*Zea mays*	Shepherd and Scott 2009

In addition to the availability of novel synthetic promoters for precise control of transgene expression the implementation of synthetic biological circuits has greatly revolutionized the prospects for plant biotechnology and modern-day agriculture. In analogy to electronic circuits, synthetic genetic circuits are the precise combinations of regulatory and coding DNA sequences introduced into crop plant for desired function. Cells respond to their environment, contribute tasks, and making decisions while the functions are controlled by DNA, RNA, and a network of interacting proteins. The ability to engineer biological circuits for a novel complex trait in plants the genetic circuit should enable different cells and tissues to process information from their surroundings and produce an appropriate response. However, the assembly of plant genetic circuits is still mostly empirical and require standardization or a precise balancing of regulator expression (Nielsen et al., 2016; de Lange et al., 2018; Kassaw et al., 2018). Equal to plant synthetic promoters the crop genetic circuit pipeline also involves different stages such as designing, assembly, and delivery into a crop plant. Thus, the quest of synthetic biology is to pave the way for designing of desired traits in different crop plants in the near future.

In early 1980s, the production of transgenic plants by *Agrobacterium*-mediated transformation initiated a new era of plant biotechnology. Presently in this review, it is difficult to review the historical developments in plant biotechnology, especially after the excellent works of Vasil, (2008) and Sussex, (2008). Whereas, modern plant biotechnology has arisen from the achievements of biochemistry and plant physiology. During the first half of the twentieth century, all key biochemical pathways were revealed, and a substantial knowledge on plant physiology had accumulated. The addition of such scientific information allowed for clear identification of most appropriate research directions as well as for better experimental designs and the field of plant biotechnology turned into a valuable tool for modern biology as predicted by Altman, (1999). In modern synthetic biology, the limitations of native or natural promoter activities such as low expression and specificity have spurred demand for the construction of synthetic promoters with higher specificity and robust expression of foreign genes. Some of the recent studies also revealed that plant synthetic promoters express genes of interest either constitutively, inductively, or in a tissue-specific manner (Chaturvedi et al., 2006; Xu et al., 2010; Hou et al., 2012; Kumar et al., 2012; Dey et al., 2015). The choice of promoter, to confer constitutive, inductive, or a tissue-specific expression is one of the key determinants and the use of synthetic promoters to assist in elucidating synergistic regulatory interactions. The role of *Cis*-regulatory elements and targeted modification of promoter architecture are necessary for coordinated manipulation of the gene activity. The identification and construction of *in-silico* promoter models has enabled more accurate prediction of gene expression, whereas the *in-silico* predictions of gene regulatory events must be validated experimentally (Novina and Roy 1996; Venter 2007).

In this article, we provide a brief description of the structure and scope of selected synthetic promoters and the engineering of their *Cis*-regulatory elements. Moreover, the functional characteristics of different synthetic promoters, their modes of regulating the expression of candidate genes in response to different conditions, and the resulting plant trait improvements reported in the past decade are discussed.

## Structure and Scope of Synthetic Promoters and the Engineering of Their *CIS*-Elements

**Figure 1:**
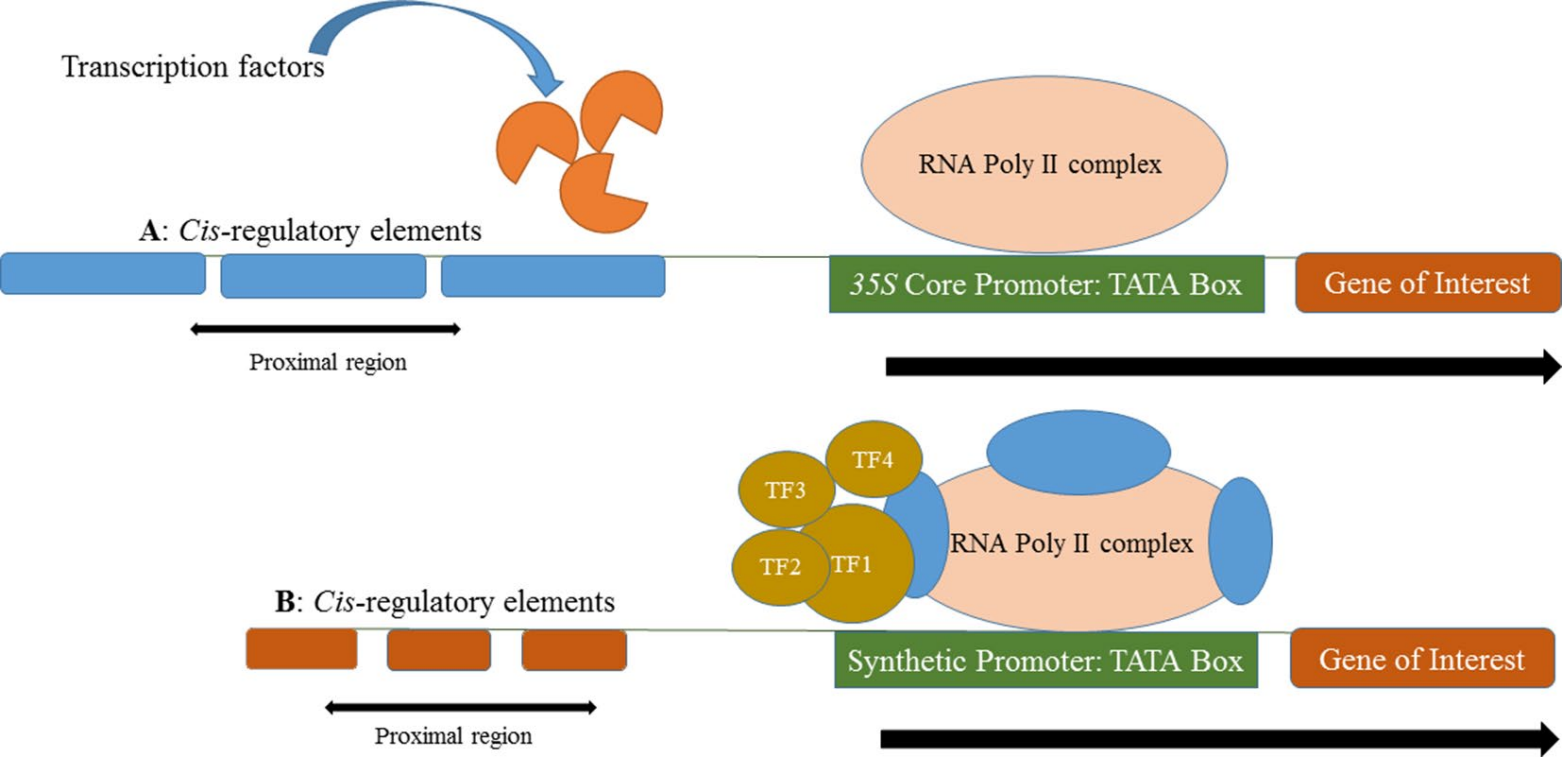
**(A)** Multiple copies of identical *cis*-regulatory elements in a long native promoter control expression of a gene of interest. Transcription factors expressed in response to the cellular environment, native *cis*-regulatory elements in the proximal region and RNA Poly II complex express the gene of interest. **(B)** A short and robust promoter in which multiple, different cis-regulatory elements from different origin that control the expression of gene of interest.

Native plant promoters are typically more than 1000 bp long and are much weaker in their expression compared to constitutive viral promoters (e.g., *CaMV 35S*) that have been commonly exploited in plant biotechnology. In contrast, synthetic promoters are typically smaller in size but generate strong constitutive or inducible expression. The primary elements (e.g., core promoter and *Cis*-motifs) of different promoters can be linked together from diverse origins to form a synthetic promoter for the spatial and temporal control of a transgene in a genetically engineered plant (**Figure 1**).

A synthetic promoter is comprised of a core promoter and synthetic *cis*-regulatory elements for the regulation of a transgene. The core promoter may be composed of an initiator region, a CAAT box, a TATA box, and GA elements (Joshi 1987; Yamamoto et al., 2011; Porto et al., 2014; Liu and Stewart 2016). The core promoter region is also known as the minimal region of the promoter, and all the core promoter elements mentioned above are not always present in each core promoter. Most commonly, the formation of a preinitiation complex takes place on the TATA box region, which is also important for the attachment of RNA polymerase II and other transcription factors (Venter 2007). In different genes the sequence analysis of core promoter elements reveals some positional conservation with respect to the transcription initiation site (TIS); this sequence is commonly present at −50 to +50 bp. The general transcription factors (GTFs) bind to the core promoter and initiate the process of transcription. The cauliflower mosaic virus (CaMV) *35S* minimal core promoter has been widely used in synthetic promoter constructs for successful expression of transgenes in plants. More recently, three switchgrass green tissue-specific minimal promoters were identified and characterized, and the expression pattern of marker genes was evaluated. Maize *Ubiquitin1* (*ZmUbi1*), switchgrass *Ubiquitin* 2 (*PvUbi2*), and *CaMV 35S* minimal promoters were used as positive controls (Liu et al., 2018).

Similarly, the *ZmUbi1* minimal core promoter from *Zea mays* was characterized for use in crop plants (Biłas et al., 2016). When constructing synthetic promoters, it is important to identify which core promoters yield the desired expression of the transgene in the recipient organism. Different core promoter elements, including the CAAT box, GA elements, and the TATA box region, can be used to construct novel and more efficient synthetic promoters. The design and engineering of core promoters along with the 5´ untranslated region (UTR) has a significant influence over the strength of transcription, initiation of translation, and the stability of the encoded gene’s mRNA. However, the presence of *cis*-regulatory modules is indispensable for the expression and specific regulation of core promoters because in the absence of *cis*-regulatory elements, core promoters yield only basal transcription or no expression (Srivastava et al., 2014; Liu and Stewart, 2016). For synthetic promoters, a generally pragmatic approach is to use *Cis*-regulatory elements that can be used with different computational tools to determine whether the appropriate binding of transcription factors is achieved and can be verified experimentally (Liu et al., 2013).

In engineering a synthetic promoter, the target is the architecture (position, nucleotide sequence, combination, and copy number) of *cis*-regulatory elements. Normally, *cis*-regulatory elements are derived from pre-existing sequences, and the choice of their copy number and spacing influences the strength and expression pattern of the synthetic promoters. Databases such as PlantCARE (Lescot et al., 2002), TRANSFAC (Matys et al., 2003), and PLACE (Higo et al., 1999) are helpful for selecting *cis*-regulatory elements with known functions, for instance enhancers or repressors. *Cis*-regulatory elements without core promoter sequences are nonfunctional and require the addition of a core promoter sequence for the attachment of transcription factors and RNA polymerase II enzymes. Recent studies on the *AOX1* promoter (*PAOX1*) in *P. pastoris* also suggested that the engineering of novel *cis*-regulatory elements is required for synthetic motif library screening, an experimental line of tracking and bioinformatics tools, because these approaches greatly affect the strength of transcription and its regulation. It was also demonstrated that there are multiple *Cis*-regulatory elements in the promoter region of *Catharanthus roseus CrWRKY1* that regulate its relevant expression. In another study they revealed that the promoter is active in both natural/native and heterologous systems, *Cis* elements can also be utilized for the isolation of regulatory factors (Yang et al., 2013).

During the past decade, several computational biology tools have been introduced for the discovery of novel *Cis*-regulatory elements. A comprehensive bioinformatics analysis of the soybean genome was conducted for *de novo* discovery of soybean cyst nematode (SCN)-inducible motifs (Liu et al., 2014). By using these bioinformatics tools, a total of 116 overlapping motif regions (OMRs) in soybean promoter *cis*-regulatory elements were identified. Tools such as SCOP, Weeder, and W-alignACE were also extremely successful for detecting core motifs (Liu et al., 2011). In addition, for developing a synthetic promoter, *Cis*-regulatory elements can be standardized because the spacing and sequence of *cis*-elements is often pivotal for inducing a helical orientation that properly interacts with the relevant transcription factors (Liu et al., 2018). Similarly, the copy number of *cis* motifs correlates with enhanced control and productivity of synthetic promoters and was confirmed in different plant species such as rice, tobacco, and *Arabidopsis* (Liu and Stewart 2016).

C*is*-regulatory elements and their respective transcription factors determine the level of transcription; signaling is generated through *cis*-*trans* interactions and directs the process of gene expression. Synthetic promoters with unique *Cis*-regulatory elements are designed to have a tailored or engineered functionality, although endogenous transcription factors and environmental stimuli can modulate functions. Homology-dependent gene silencing is a concern that can be avoided by designing synthetic promoters with minimal sequence similarity to the recipient cells’ genome (Venter and Botha 2010). In this manner, a detailed method for the identification of *cis*-regulatory elements that function in the promoter of germline-expressed genes and display a unique transcriptional profile has been described (Peters et al., 2017). More recently, it has been proposed that combinatorial *cis-trans* engineering for the introduction of specific traits into plants also enhances environmental adaptability (Shrestha et al., 2018). A CRISPR*-dCas9*-based bipartite module was used to regulate the expression of multiple target genes (Shrestha et al., 2018). The construction of combinatorial promoter libraries and other computational tools has the potential to contribute to the engineering of different *Cis*-regulatory elements for synthetic promoters. Moreover, the use of synthetic promoters in combination with bioinformatics tools, such as the binding-site estimation suite of tools (BEST) platform, could become a powerful approach for the evaluation and discovery of novel *cis*-sequences in response to plant-pathogen interactions or any other specific stimuli (Koschmann et al., 2012).

## Functional Characteristics of the Promoters and Transgenic Plants

The different types of promoters are classified based on gene expression and their regulation. The most common types of gene expression are constitutive, inducible, and tissue specific. In constitutive expression, a promoter may be active throughout all developmental stages of the plant in each tissue. In contrast, an inducible promoter is modulated by external stimuli such as different biotic and abiotic environmental factors. Inducible promoters are also expressed at specific developmental stages without endogenous factors. Tissue-specific promoters direct the expression of a gene in one or more tissues or at certain stages of development. Successful regulation of promoter activity is achieved by coordinated expression of transcription factors; hence, the activity of promoters from monocotyledonous plants is higher in monocots compared to dicots (Cornejo et al., 1993; Park et al., 2010; Biłas et al., 2016). However, synthetic promoters are designed by bringing together promoter elements from diverse origins to achieve the desired type of expression. There are various sets of regulatory elements in different promoters that can be reorganized and ligated with synthetic and natural *cis* sequences to construct synthetic promoters. To study *cis* elements and promoter structures, two approaches, deconstructive and reconstructive, have been reported (Mehrotra et al., 2011). The deconstructive approach involves removing nucleotides or introducing mutations in the nucleotide sequence of *cis* elements. The reconstructive approach involves the addition of specific *cis*-regulatory elements to minimal promoter sequences. Both approaches can lead to the development of novel synthetic promoters (Bhullar et al., 2003; Venter 2007; Mehrotra et al., 2011).

### Constitutive Promoters and Constitutive Expression of Synthetic Promoters

Early endeavors in plant biotechnology, constitutive promoters were isolated from plant pathogens (such as cauliflower mosaic virus; *CaMV 35S*) and used for the expression of transgenes in plants (Odell et al., 1985; Benfey et al., 1990a; Benfey and Chua, 1990b). These promoters are active throughout almost all developmental stages and tissue in the plant and are minimally affected by environmental factors (**Table 1**). One of the prominent characteristics of constitutive promoters is their activity and expression across different species and even kingdoms (Benfey et al., 1989). The constitutive expression of reporter proteins, transcription factors, and the production of bioactive compounds at all stages of plant growth and development have used constitutive promoters in different expression vectors. However, constitutive promoters isolated from plant pathogens can lead to abnormal conditions in transgenic plants. Therefore, constitutive promoters of plant origin have been isolated, such as the moderate constitutive promoter (*PtMCP)* isolated from *Populus tomentosa; PvUbi1* and *PvUbi2* isolated from switchgrass; and *CsCYP*, *C2* (*CsGAPC2*), and *CsEF1* isolated from citrus (Mann et al., 2011; Chen et al., 2013; Erpen et al., 2018).

Currently, these plant-based constitutive promoters are preferred over pathogen-derived constitutive promoters (Potenza et al., 2004), but there are still some exceptions to the constitutive overexpression of promoters. For instance, constitutive overexpression of genes related to defense mechanisms may cause increased disease resistance but may also lead to stunted plant growth of or disease symptoms, even in the absence of plant pathogens (Fitzgerald et al., 2004; Venter 2007). The expression of natural promoters can also be downregulated after they are cloned into an expression construct. For example, the constitutive promoter (*Act2*) of *Arabidopsis* failed to enhance protein expression in microsporangia or seed coats of *Arabidopsis*, and the modified actin promoter of rice did not cause xylem expression in rice plants (Zhang et al., 1991; An et al., 1996; Biłas et al., 2016). Moreover, bidirectional promoter constructs were also used to constitutively express two transgenes, in which two unidirectional promoters were joined in opposite directions, but the expression failed to reach the expected level (Zhang et al., 2008). These findings have increased interest in the design of synthetic promoters that drive constitutive expression.

Most plant promoters are comprised of a core set of elements: a cap, or transcription start, site; a CCAAT consensus sequence; and a TATA box. Generally, synthetic promoters are designed by joining the upstream activation sequence of one promoter to a TATA box-containing region of another promoter for constitutive expression of the transgene. The pioneering design of the first synthetic promoter paved the way for designing other promoters (Comai et al., 1990). A hybrid promoter, known as “Mac,” in which the *Ti* plasmid mannopine synthase promoter (+65 to −301) was incorporated into the enhancer region (−90 to −941) of *CaMV 35S*, yielded interesting results. The Mac promoter overexpressed *GUS* compared to double *CaMV 35S* promoters in the leaves and roots (Comai et al., 1990). In another study, two constitutive promoters (*CaMV 35S* and *Actin-1* from rice) with a compact chimeric *Bt* gene and *cryIA(b)* of *Bacillus thuringiensis* were joined (Datta et al., 1998). The authors reported 100% insect larvae mortality in 81 transgenic plants (Datta et al., 1998). Subsequently, the *in vivo* gene expression and regulation properties of a G-box motif with different flanking sequences were investigated (Ishige et al., 1999). *GUS* gene expression driven by 11 different G-box tetramers fused to a promoter (*CaMV* ± 90/*35S*) was studied in transgenic tobacco plants (Ishige et al., 1999). The G-box-10 construct conferred high-level constitutive expression in different parts of transgenic dicot (carrot) and monocot (rice) plants (Ishige et al., 1999).

To study the expression of functionally identical promoters having minimal sequence homology in transgenic plants (Bhullar et al., 2003), two synthetic promoters, *Mod2A1T* and *Mod3A1T*, were constructed by placing the minimal promoter and sub-domain A1 core elements into divergent DNA sequences. The activity of these synthetic promoters was comparable to that of the *CaMV 35S* promoter in different tissues of transgenic *Nicotiana tabacum*. This strategy revealed the possibility of designing synthetic promoters having minimal sequence homology by modifying sequences between *cis*-elements for transgene expression (Bhullar et al., 2003).

Short repeated DNA enhancer elements of viral origin increased the expression of genes when they were combined with the minimal *CaMV 35S* promoter. Synthetic promoters with multiple copy numbers, in combination with direct repeat cassettes, caused constitutive expression of the reporter gene (*luciferase*) in an *Agrobacterium*-based leaf-infiltration transient assay in *Nicotiana tabacum* (Cazzonelli and Velten 2008). Higher constitutive expression levels of different synthetic promoters and their increased translation properties in both transient and transgenic assays have also been reported (Wever et al., 2010). Three synthetic promoter constructs were designed by using three different 5´ UTRs of *Vigna radiate* aminocyclopropane-1-carboxylate synthase (*VR-ACS1*) gene coupled with a *CaMV 35S* promoter and β-glucuronidase (*GUS*) reporter gene (Wever et al., 2010). The transgenic seedlings and mature plants with 5´ UTRs from the *VR-ACS1* and *Cab22L* genes showed a two- to five-fold increase in GUS activity (Wever et al., 2010).

Kumar et al. (2011) designed a strong recombinant promoter (*MSgt-FSgt*) for high level expression of transgenes in different plants. Ten recombinant promoters with different upstream activation sequences were generated by incorporating the *Mirabilis mosaic* virus sub-genomic transcript (MS8, −306 to +27) and TATA-containing core domains of the *Figwort mosaic* virus sub-genomic transcript promoter (FS3, −271 to +31). The successful regulation of recombinant promoters for *GUS* and *GFP* was confirmed in tobacco protoplasts. The engineered promoters revealed stronger activity compared to the *CaMV 35S* constitutive promoter (Kumar et al., 2011).

Synthetic promoters where *cis* elements are shuffled can be advantageous in developing transgenic plants that express the trait of interest. Acharya et al. (2014a) developed a constitutive synthetic promoter (*MUSASMSCP*) by incorporating the upstream activation sequence of *Mirabilis mosaic* virus (MMV) full-length transcript (−279 to −38) to the 5´end of the MMV sub-genomic transcript (−306 to −125) promoter fragment. Subsequently, the transient activity of the *MUASMSCP* promoter was evaluated in *Petunia hybrida* and tobacco protoplasts and showed constitutive expression of the transgenes at levels comparable to that of the *CaMV 35S* promoter (Acharya et al., 2014a). In another study, a set of three chimeric/hybrid promoters (*FSgt-PFlt, PFltUAS-2X*, and *MSgt-PFlt*) were developed, and each chimeric/hybrid promoter drove the constitutive expression of multiple reporter genes in different plant species, including tobacco, spinach, *Arabidopsis*, petunia, and tomato. In addition, promoter activity was induced by salicylic acid and abscisic acid (Acharya et al., 2014b).

Another study used different *cis* elements to generate synthetic promoters, *SynS1* and *SynS2*, which conferred constitutive *GUS* expression to the stems of transgenic sugarcane (Chakravarthi et al., 2015). Peramuna et al. (2018) tested three (12–10, 12–48, 12–79) synthetic promoters in *Physcomitrella patens*. These short synthetic promoters showed higher mRNA expression than the endogenous *PpAct7* promoter, with protein activity similar to that of the medium-strength *AtUBQ10* promoter. The authors also suggested a strategy for constructing multiple synthetic promoters on demand for other plants (Peramuna et al., 2018). All these studies demonstrate that the use of constitutive synthetic promoters to produce targeted proteins or metabolites in greater amounts will contribute to the design of genetically modified crop plants and to modern agriculture at large.

### Inducible Promoters and Inducible Expression of Synthetic Promoters

A promoter whose activity is triggered by the presence or absence of biotic or abiotic factors is known as an inducible promoter. The controllable expression of inducible promoters is of great importance in gene expression and recombinant DNA technology. In genetic engineering of plants, inducible promoters are frequently used because the gene of interest can be switched off or on under certain conditions or at certain developmental stages. Several different inducible promoters have been reported that vary according to their source and expression level. Inducible promoters are broadly grouped as either being chemically or physically regulated. A promoter whose expression is regulated by the availability of chemicals such as steroids, metals, hormones, or alcohols, is called chemically inducible (**Table 2**). A promoter whose transcriptional activity is regulated by plant pathogens, temperature, or light levels is called physically inducible (Padidam 2003; Boni et al., 2018). In transgenic plants, the expression of inducible promoters in response to various plant pathogens provides broad-spectrum, pathogen-inducible resistance. Broadly, a plant pathogen inducible promoter has to be a chemically inducible promoter because it is not the pathogen itself that causes induction but chemical substances like MAMPs or elicitors that execute the induction. Even if it would be the pathogen it would still be a chemical substance.

Similarly, the promoters should not express their transgenes under normal conditions, ensuring that no defense responses are needlessly activated (Shehzadi et al., 2018). Boni et al. (2018) reported a *Ta-Lr34res* gene that encodes an ATP-binding cassette (ABC) transporter protein that confers broad-spectrum resistance against fungal pathogens in wheat plants at adult stages. *Ta-Lr34res* that was expressed under the control of the pathogen-inducible *Hv-Ger4c* promoter in transgenic barley conferred enhanced resistance against powdery mildew and leaf rust, even at the seedling stage. These results suggested that *Ta-Lr34res* driven by the pathogen-inducible *Hv-Ger4c* promoter is agronomically useful in barley, and that the negative effects of the endogenous promoter can be eliminated without compromising disease resistance (Boni et al., 2018).

Various chemically-inducible promoters that initiate the expression or suppression of a gene at a defined time during plant development have also been reported. However, the spraying of such chemicals may not reach all the infection sites, providing only short-lived control of disease, which can lead to undesirable effects on the plant tissues (Zuo and Chua 2000; Deveaux et al., 2003; Gurr and Rushton 2005). Kinkema et al. (2014) *used a modified alc* gene expression system that was naturally ineffective in monocots (e.g., sugarcane). A promoter consisting of tandem copies of the ethanol receptor inverted repeat binding site was used in combination with a minimal promoter sequence to yield an ethanol-inducible promoter. This promoter conferred enhanced sensitivity and significantly higher expression levels in a modified *alc* gene expression system (Kinkema et al., 2014). Similarly, a strong oxidative stress-inducible promoter (POD) was cloned by Kim et al. (2003) from sweet potato, while the study of Zhu et al. (2015) revealed a synthetic probenazole (PBZ)-inducible promoter by combining different *cis-*elements, and its expression pattern in response to multiple signaling pathways such as salicylic acid, jasmonic acid, ethylene, calcium, and mitogen-activated protein kinase (MAPK) was evaluated in *Arabidopsis* (Zhu et al., 2015). Recently, certain chemically- or pathogen-inducible promoters showed background expression levels in different transgenic plants, making them incompatible tools for biotechnological applications that require inducible expression; however, these results support the development of inducible synthetic promoters in transgenic plants.

Hou et al. (2012) reported the development of three inducible synthetic promoters, *EKCM, EKCRM*, and *ECCRM*, which consist of multiple *cis*-acting elements that respond to cold and salt stresses and drive stress-inducible transgene expression with minimal negative effects on transgenic *Arabidopsis thaliana*. Each promoter was independently linked to the *β-glucuronidase* (*GUS*) reporter gene, and GUS expression was analyzed in *A. thaliana*. GUS activity under stress was significantly higher in transgenic plants with the synthetic promoter constructs than in plants with the control construct where *GUS* expression was driven by the responsive to dehydration 29A (*rd29A*) promoter. In particular, *EKCM* showed a 1.29-fold increase in GUS activity in different tissues of transgenic *A. thaliana* under dehydration conditions (Hou et al., 2012). Synthetic promoters Ap, Dp, and ANDp were also designed to provide drought resistance in plants (Ge et al., 2018). The combination of functional genes for cytosolic abscisic acid (ABA) receptor kinase 1 (*CARK1*) and regulatory components of ABA receptor 11 (*RCAR11*) with synthetic promoters resulted in drought stress tolerance. It was also reported that exogenous ABA or co-transformation with the effector dehydration-responsive element binding protein 2A (DREB2A) induced the activity of the synthetic promoters (Ge et al., 2018). They concluded that a combination of synthetic promoters and functional genes (*Dp.RCAR11*-*Ap.CARK1* and *ANDp.CARK1*) can be used to generate drought stress resistance in different plants.

To construct synthetic pathogen-inducible promoters, Shokouhifar et al. (2011) used two known pathogen-inducible *cis*-acting elements, F and E17, in dimers and in combination, and placed them upstream of the minimal *CaMV 35S* promoter. The resulting constructs, pGEE, pGFF, and pGFFEE, were analyzed after transformation into canola (*Brassica napus) plants*. GUS histochemical analyses revealed that the synthetic promoters responded to phytohormone treatments and fungal elicitors (Shokouhifar et al., 2011).

Moradyar et al. (2016) reported that the synthetic pathogen-inducible promoter SP-DDEE, containing parsley D, E17 elements, and a minimal promoter, strongly halted the growth of the phytopathogenic fungus *Sclerotinia sclerotiorum*, showing again how important inducible synthetic promoters can be for controlling the expression of genes related to plant defense mechanisms and engineering plants with increased resistance to pathogens and other environmental stressors.

### Tissue-Specific Promoters and Tissue-Specific Expression of Synthetic Promoters

A promoter whose activity is observed only in specific tissues of the plant is known as a tissue-specific promoter. Such promoters express the cistronic part of their gene in cells of particular tissue types, and their expression may also be induced in those tissues by internal or external factors. A transgene ligated to a tissue-specific promoter should activate expression of the encoded protein in a particular cell type, without affecting unmodified tissues of the plant. Recently, a variety of tissue-specific promoters have been reported from plant tissues, and studies have shown that their expression provides an advantage over constitutive promoters because it is based on the interacting levels of gene expression and regulation (**Table 3**). Tissue-specific expression of transgenes will often require the use of promoters from closely related plant species.

In the last decade, several root-, seed-, chloroplast-, and fruit-specific promoters have been characterized. The root-specific expression of aminocyclopropane-1-carboxylate (ACC) deaminase under the control of the root locus *rolD* promoter from *Agrobacterium rhizogenes* was evaluated in transgenic tomato plants (Grichko et al., 2005). Koramutla et al. (2016) reported the strength and stability of expression driven by *cis* motifs identified through *in silico* screening, one of which was the phloem-specific promoter for *Glutamine synthetase 3A* (*GS3A*) gene, in *Brassica juncea* phloem exudates. A number of different seed-specific promoters have also been identified for the purposes of enhancing the nutritive value of rice, corn, and legumes. Promoters active in seeds, such as the glutelin promoter in rice, the zein promoter in maize, and the arcelin (*arc5-I*) promoter in the bean *Phaseolus vulgaris*, were able to transform and greatly improve the nutritive value of seeds with heterologous proteins (De Jaeger et al., 2002; Sharma and Sharma 2009). Recently, specific promoters have been used to express foreign proteins in chloroplasts. The 16S rRNA promoter has been repeatedly used to transform chloroplasts. Promoters such as *Prrn* and *psbA* have been used to transform tobacco chloroplasts for the production of insulin (*CTB‐Pins*) and other protective antigens (Koya et al., 2005; Ruhlman et al., 2007). One area of interest is to use fruit-specific promoters to enhance the nutritive value and flavor of fruits or to produce edible vaccines. Fruits of genetically engineered plants offer an economical and harmless source for vaccine development, including cholera toxin B (CTB) vaccines. The tomato fruit-specific *E8* promoter has been tested for expressing antigens in fruits, and these tests provided a basis for exploring the use of CTB expression in tomato fruits as edible vaccines (Jiang et al., 2007).

Although constitutive promoters have been used in transgenic studies, they carry the risk of unwanted or off-target effects, such as the silencing of epigenetic genes and suboptimal growth. However, the use of tissue-specific promoters for targeted transgene expression can potentially overcome these problems. Initially, the function of plant heat shock promoter elements in the expression of chimeric genes was analyzed (Schöffl et al., 1989). A series of mutations were introduced into different portions of the promoters of heat shock genes in soybean and then linked to chloramphenicol acetyl transferase coding sequences for expression in tobacco. This study revealed that the TATA box of heat shock promoter elements (HSEs) can be substituted by other native and overlapping HSE-containing upstream sequences, while synthetic promoter elements could also be used (Schöffl et al., 1989). Lam and Chua (1991) used the *hex-3* and *hex-1* elements of the wheat histone gene promoter construct synthetic promoters *4H3-46* and *4H1-46*. Only a tetramer of *hex-3* in transgenic tobacco was shown to overexpress the target gene upon exposure to saline (NaCl) and elevated concentrations of ABA (Lam and Chua 1991). Clendennen et al. (1999) designed a synthetic promoter to downregulate the expression of the S-adenosylmethionine-cleaving enzyme (SAMase) in the ripening fruit of cantaloupe. Similarly, an *E8-E4* hybrid synthetic promoter, composed of *cis*-acting elements derived from the *E8* and *E4* genes of tomato, has been patented to enhance expression of foreign genes in fruits; could be used to reduce the production of ethylene, which has a pivotal role in fruit ripening (Bestwick and Kellogg 2000).

In the last decade, several studies have reported various synthetically engineered, tissue-specific promoters for the successful expression of genes of interest. A *Zea mays* chimeric promoter having activity specific to the kernel endosperm and embryo was constructed and evaluated (Shepherd and Scott 2009). The authors combined the elements of the *27zn* and *Glb1* promoters to construct a synthetic chimeric promoter *A27znGlb1*. This newly designed promoter showed the capability for tissue-specific expression in the embryo and kernel endosperm of maize transgenic plants (Shepherd and Scott 2009). Li et al. (2013) produced a pCL synthetic promoter by fusing two DNA cassettes (containing the *Arabidopsis cor15a* promoter and the potato *patatin* promoter regions) that specifically regulates the activity of acid vacuolar invertase in potato tubers at low temperature, but has no effect in other tissues or in tubers under normal conditions. Transgenic potato lines were constructed with a vector containing 955 bp of antisense *StvacINV1* sequence under the control of the pCL promoter to confirm the function of the pCL promoter in cold-induced sweetening (CIS) in potato. These results demonstrated the utility of using the pCL promoter to prevent CIS in potato tubers (Li et al., 2013). Du et al. (2014) constructed four different chimeric promoters (p35S-*LCHS*-Ω, pOCS-*PCHS*-Ω, pOCS-*LCHS*-Ω, and p35S-*PCHS*-Ω) in a quest for a better-quality flower-specific promoter. The study combined *CaMV 35S* or the *OCS* enhancer with a *CHSA* core promoter fragment, from either petunia or lily, along with an omega (Ω) element and evaluated the levels and tissue specificity of *GUS* expression in transgenic *Torenia fournieri*. Among these chimeric promoters, pOCS-*PCHS*-Ω showed high expression levels in colored corollas (Du et al., 2014).

Synthetic biology has been applied in different fields, and it has revolutionized plant biotechnology. The study of Wang et al. (2015) provides a brilliant example of the synthesis of tissue-specific promoters, and it presents a practical method for genome-wide screening and functional identification of tissue-specific *Cis* elements in rice. Five novel tissue-specific promoters were synthesized, and they revealed green tissue-specific *cis* elements that showed different expression levels in various green tissues of rice (Wang et al., 2015). Moreover, a novel synthetic root-specific promoter (*SynR2*) was constructed and evaluated in transgenic tobacco plants (Mohan et al., 2017). In a very recent study, Bai et al. (2019) revealed four synthetic promoters (*BiGSSP2, BiGSSP3, BiGSSP6*, and *BiGSSP7*) with high bidirectional expression efficiencies specifically in green tissues, which can be widely applied to agriculture biotechnology. Furthermore, bidirectional green tissue‐specific promoters have important application prospects in genetic engineering and crop genetic improvement. However, there is no report on the application of them, mainly due to undiscovered natural bidirectional green tissue‐specific promoters and the lack of a comprehensive approach for the synthesis of these promoters (Bai et al., 2019). Similarly, the application of seed-specific bidirectional promoters for the metabolic engineering of anthocyanin-rich crop plants was reported (Liu et al., 2018).

## Synthetic Transcriptional Tools and Promoters

The development and use of synthetic promoters for transgene expression has increased interest in controlled regulatory mechanisms for the prolific expression of genes of interest. Not only do *cis*-regulatory elements such as proximal elements and core promoter region control the expression and regulation of transgenes in plants, but elements such as silencers, insulators, and enhancers along with their associated transcription factors also play a prominent role (Spitz and Furlong 2012). Like native promoters, synthetic promoters also require appropriate transcriptional tools, such as activators and repressors, for the precise regulation of transgenes (Liu et al., 2013; Petolino and Davies 2013). According to Liu and Stewart (2016), different synthetic transcription factors can be designed by using fusion proteins consisting of DNA binding domains (DBDs) with effector domains to activate or repress a transgene. Activation domains, such as VP16 from herpes simplex virus, assist the attachment of different transcription factors (e.g., TFIID, TFIIH) to the promoter region for the formation of preinitiation complexes in plants. However, repressor domains partially halt or suppress the expression of genes by using chromatin remodeling factors (CRFs); one of the best examples is EAR and its derivative SRDX domains in tobacco plants (Hiratsu et al., 2003; Hirai et al., 2010; Mahfouz et al., 2012; Liu and Stewart 2016).

Different approaches have been used to target gene activation or to target genome modification in plants. The use of synthetic transcription factors for targeted gene activation (e.g., zinc-finger transcription factors, ZF-TFS; transcription activator-like effector transcription factors, TALE-TFs) or enzymes for genome modification (e.g., zinc finger nucleases, ZFNs; TALE nucleases, TALENs) will obviously facilitate metabolic engineering in plant synthetic biology. The activation domains of synthetic ZF-TFs have been used for targeted activation of genes in *Brassica napus*, such as the β‐ketoacyl‐acyl-carrier-protein synthase II (*KASII*) gene (Guan et al., 2002), and the tissue-specific promoter from *APETALA1* in *Arabidopsis thaliana* (Gupta et al., 2012). These studies also found that synthetic transcription factors based on zinc finger proteins (ZFPs) are capable of stably regulating endogenous genes for stimulating the expression of specific agronomically relevant traits through several crop cycles.

Mahfouz et al. (2012) reported that TALEs contain a modular DNA-binding domain that can be easily engineered to bind to a specific sequence, and the use of TALEs for the generation of chimeric sequence-specific transcriptional repressors has been demonstrated. Targeted repression was confirmed in *A. thaliana* after salt and cold treatment when TALE DNA-binding domains were fused to the EAR-associated repression domain (SRDX) for both endogenous and transgenic *RD29A* promoter expression (Mahfouz et al., 2012). The application of sequence-specific transcriptional repression tools can be used to assess functional genomics in detail and other related biotechnological applications. Furthermore, TALE-TFs and ZF-TF tools can be used to activate key regulatory proteins and to effect plant genome editing during transformation. However, these techniques will need vigilant planning and analysis to ensure the appropriate specificity and efficiency required for suitable production. Synthetic transcription factors and synthetic promoters could be paired for robust expression of a transgene and the synthesis of its encoded product in plants. Therefore, these are advantageous approaches for gene expression, gene regulation, crop improvement, and sustainable agricultural production.

## Conclusion and Future Perspective

In recent decades, sustainable agriculture production has become an urgent issue from the perspectives of food security and global climate change. The application of plant biotechnology tools for the development of crop plants that are better adapted to stressful conditions is important for more sustainable production and harvesting greater quantities of food (Varshney et al., 2011). Various model and crop plants have been genetically modified to improve traits and to produce bioactive secondary metabolites. However, further progress in crop improvement and plant biotechnology still requires tools that are more precise than those currently available. The application of synthetic biology for the rearrangement of genetic components that are found in nature has revolutionized the field of plant molecular biotechnology. Since its inception more than a decade ago, the field of synthetic biology has grown significantly and has generated several prominent developments, such as the use of synthetic promoters and synthetic transcriptional tools to replace native/natural promoters in plant biotechnology.

One goal in plant biotechnology is to successfully express multiple transgenes in a single transgenic plant to enhance agricultural productivity and consequently contribute toward feeding the population of nine billion people estimated for 2050 (Godfray et al., 2010; Tomlinson 2013). Independent expression systems and novel regulatory components could be engineered for genes of interest, which could be fruitful for expressing desired traits or metabolic activity in plants. Thus, synthetic promoters are valuable tools for the genetic modification of plants, and they have potential for making meaningful contributions to the improvement of the agricultural sector at large. The use of synthetic promoters can not only enhance agricultural productivity under diverse environmental conditions but could also be useful for producing pharmaceutical products inside plant tissues. Therefore, synthetic promoters that result in a desired level of expression of their associated transgenes are important, as are the core promoter and *cis*-regulatory elements that have already been designed and studied over the past decade.

In this review, we surveyed the structure, synthesis, and expression of synthetic promoters reported in the preceding decade. Parameters such as specificities and strength were chosen to categorize the reviewed promoters, which included synthetic constitutive promoters, synthetic inducible promoters, and synthetic tissue-specific promoters (**Tables 1**–**3**). Such a classification scheme for the description of synthetic promoters and their *Cis*-regulatory elements will greatly facilitate the selection of relevant regulatory components for future research. Furthermore, it will also enhance our understanding for developing transgenic plants that are resistant to multiple stress conditions. Besides synthetic promoters, other synthetic biology tools (ZF-TF, TALENs) and systems (synthetic genomes, synthetic biological circuits) have been designed; their application in crop plants will definitely enhance our understanding for increasing the availability of desired products. The combined use of synthetic promoters and synthetic transcription tools have the capability to modernize plant genetic modification and genetic technology. Similarly, the use of tools, such as CRISPR-*Cas*, RNA*i*, and other plant genomic techniques, will soon revolutionize the field of plant biotechnology, enabling precise and targeted genome editing and the development of novel plant species with desired traits.

## Author Contributions

SA and WK have made substantial, direct, and intellectual contribution to conceptualize the review outline and wrote the manuscript.

## Conflict of Interest

The authors declare that the research was conducted in the absence of any commercial or financial relationships that could be construed as a potential conflict of interest.

## References

[B1] Acharya S. Ranjan R. Pattanaik S. Maiti I. B. Dey N. (2014b). Efficient chimeric plant promoters derived from plant infecting viral promoter sequences. Planta 239 (2), 381–396. 10.1007/s00425-013-1973-2 24178585

[B2] Acharya S. Sengupta S. Patro S. Purohit S. Samal S. K. Maiti I. B. (2014a). Development of an intra-molecularly shuffled efficient chimeric plant promoter from plant infecting Mirabilis mosaic virus promoter sequence. J. Biotechnol. 169, 103–111. 10.1016/j.jbiotec.2013.08.022 24060830

[B3] Altman A. (1999). Plant biotechnology in the 21st century: the challenges ahead. Electronic J Biotechnol (www.ejb.org). 10.2225/vol2-issue2-fulltext-1

[B4] An Y. Q. McDowell J. M. Huang S. McKinney E. C. Chambliss S. Meagher R. B. (1996). Strong, constitutive expression of the Arabidopsis ACT2/ACT8 actin subclass in vegetative tissues. Plant J. 10 (1), 107–121. 10.1046/j.1365-313X.1996.10010107.x 8758981

[B5] Bai J. Wang X. Wu H. Ling F. Zhao Y. Lin Y. (2019). Comprehensive construction strategy of bidirectional green-tissue specific synthetic promoters. Plant. Biotechnol. J., 213–231. 10.1111/pbi.13231 PMC7004895 31393049

[B6] Benfey P. N. Chua N. H. (1990b). The cauliflower mosaic virus 35S promoter: combinatorial regulation of transcription in plants. Science 250 (4983), 959–966. 10.1126/science.250.4983.959 17746920

[B7] Benfey P. N. Ren L. Chua N. H. (1989). The CaMV 35S enhancer contains at least two domains which can confer different developmental and tissue-specific expression patterns. EMBO J. 8 (8), 2195–2202. 10.1002/j.1460-2075.1989.tb08342.x 16453896 PMC401147

[B8] Benfey P. N. Ren L. Chua N. H. (1990a). Tissue-specific expression from CaMV 35S enhancer subdomains in early stages of plant development. EMBO J. 9 (6), 1677–1684. 10.1002/j.1460-2075.1990.tb08291.x 2347301 PMC551870

[B9] Bestwick R. K. Kellogg J. A., (2000). U.S. Patent No. 6,118,049. Washington, DC: U.S. Patent and Trademark Office.

[B10] Bhullar S. Chakravarthy S. Advani S. Datta S. Pental D. Burma P. K. (2003). Strategies for development of functionally equivalent promoters with minimum sequence homology for transgene expression in plants: cis-elements in a novel DNA context versus domain swapping. Plant Physiol. 132 (2), 988–998. 10.1104/pp.103.020602 12805627 PMC167037

[B11] Biłas R. Szafran K. Hnatuszko-Konka K. Kononowicz A. K. (2016). Cis-regulatory elements used to control gene expression in plants. Plant Cell Tissue Organ Cult. (PCTOC) 127 (2), 269–287. 10.1007/s11240-016-1057-7

[B12] Boni R. Chauhan H. Hensel G. Roulin A. Sucher J. Kumlehn J. (2018). Pathogen-inducible Ta-Lr34res expression in heterologous barley confers disease resistance without negative pleiotropic effects. Plant Biotechnol. J. 16 (1), 245–253. 10.1111/pbi.12765 28561994 PMC5785347

[B13] Cazzonelli C. I. Velten J. (2008). In vivo characterization of plant promoter element interaction using synthetic promoters. Transgenic Res. 17 (3), 437–457. 10.1007/s11248-007-9117-8 17653610

[B14] Chakravarthi M. Narayan J. A. Subramonian N. Appunu C. (2015). Development of novel synthetic promoters for gene expression in transgenic sugarcane. J. Sugarcane Res. 5 (2), 42–52.

[B15] Chaturvedi C. P. Sawant S. V. Kiran K. Mehrotra R. Lodhi N. Ansari S. A. (2006). Analysis of polarity in the expression from a multifactorial bidirectional promoter designed for high-level expression of transgenes in plants. J. Biotechnol. 123 (1), 1–12. 10.1016/j.jbiotec.2005.10.014 16324763

[B16] Chen Z. Wang J. Ye M. X. Li H. Ji L. X. Li Y. (2013). A novel moderate constitutive promoter derived from poplar (Populus tomentosa Carrière). Int. J. Mol. Sci. 14 (3), 6187–6204. 10.3390/ijms14036187 23507754 PMC3634493

[B17] Clendennen S. K. Kellogg J. A. Wolff K. A. Matsumura W. Peters S. Vanwinkle J. E. (1999). “Genetic engineering of cantaloupe to reduce ethylene biosynthesis and control ripening,” in Biology and Biotechnology of the Plant Hormone Ethylene II (Dordrecht: Springer), 371–379. 10.1007/978-94-011-4453-7_68

[B18] Comai L. Moran P. Maslyar D. (1990). Novel and useful properties of a chimeric plant promoter combining CaMV 35S and MAS elements. Plant Mol. Biol. 15 (3), 373–381. 10.1007/BF00019155 2103458

[B19] Cominelli E. Galbiati M. Albertini A. Fornara F. Conti L. Coupland G. (2011). DOF-binding sites additively contribute to guard cell-specificity of AtMYB60 promoter. BMC Plant Biol. 11 (1), 162. 10.1186/1471-2229-11-162 22088138 PMC3248575

[B20] Cornejo M. J. Luth D. Blankenship K. M. Anderson O. D. Blechl A. E. (1993). Activity of a maize ubiquitin promoter in transgenic rice. Plant Mol. Biol. 23 (3), 567–581. 10.1007/BF00019304 8219091

[B21] Datta K. Vasquez A. Tu J. Torrizo L. Alam M. F. Oliva N. (1998). Constitutive and tissue-specific differential expression of the cryIA (b) gene in transgenic rice plants conferring resistance to rice insect pest. Theor. Appl. Genet. 97 (1–2), 20–30. 10.1007/s001220050862

[B22] De Jaeger G. Scheffer S. Jacobs A. Zambre M. Zobell O. Goossens A. (2002). Boosting heterologous protein production in transgenic dicotyledonous seeds using Phaseolus vulgaris regulatory sequences. Nat. Biotechnol. 20 (12), 1265. 10.1038/nbt755 12415287

[B23] de Lange O. Klavins E. Nemhauser J. (2018). Synthetic genetic circuits in crop plants. Curr. Opin. Biotechnol. 49, 16–22. 10.1016/j.copbio.2017.07.003 28772191 PMC6007868

[B24] Deveaux Y. Peaucelle A. Roberts G. R. Coen E. Simon R. Mizukami Y. (2003). The ethanol switch: a tool for tissue-specific gene induction during plant development. Plant J. 36 (6), 918–930. 10.1046/j.1365-313X.2003.01922.x 14675455

[B25] Dey N. Sarkar S. Acharya S. Maiti I. B. (2015). Synthetic promoters in planta. Planta 242 (5), 1077–1094. 10.1007/s00425-015-2377-2 26250538

[B26] Du L. Lou Q. Zhang X. Jiao S. Liu Y. Wang Y. (2014). Construction of flower-specific chimeric promoters and analysis of their activities in transgenic torenia. Plant Mol. Biol. Rep. 32 (1), 234–245. 10.1007/s11105-013-0646-4

[B27] Erpen L. Tavano E. C. R. Harakava R. Dutt M. Grosser J. W. Piedade S. M. S. (2018). Isolation, characterization, and evaluation of three Citrus sinensis-derived constitutive gene promoters. Plant Cell Rep. 37 (8), 1113–1125. 10.1007/s00299-018-2298-1 29796947

[B28] Fitzgerald H. A. Chern M. S. Navarre R. Ronald P. C. (2004). Overexpression of (At) NPR1 in rice leads to a BTH-and environment-induced lesion-mimic/cell death phenotype. Mol. Plant-Microbe Interact. 17 (2), 140–151. 10.1094/MPMI.2004.17.2.140 14964528

[B29] Gasser C. S. Fraley R. T. (1989). Genetically engineering plants for crop improvement. Science 244 (4910), 1293–1299. 10.1126/science.244.4910.1293 17820660

[B30] Ge H. Li X. Chen S. Zhang M. Liu Z. Wang J. (2018). The Expression of CARK1 or RCAR11 Driven by Synthetic Promoters Increases Drought Tolerance in Arabidopsis thaliana. Int. J. Mol. Sci. 19 (7), 1945. 10.3390/ijms19071945 PMC6073707 29970817

[B31] Gerasymenko I. M. Sheludko Y. V. (2017). Synthetic cold-inducible promoter enhances recombinant protein accumulation during Agrobacterium-mediated transient expression in Nicotiana excelsior at chilling temperatures. Biotechnol. Lett. 39 (7), 1059–1067. 10.1007/s10529-017-2336-z 28439740

[B32] Godfray H. C. J. Beddington J. R. Crute I. R. Haddad L. Lawrence D. Muir J. F. (2010). Food security: the challenge of feeding 9 billion people. Science 327 (5967), 812–818. 10.1126/science.1185383 20110467

[B33] Goodman R. M. Hauptli H. Crossway A. Knauf V. C. (1987). Gene transfer in crop improvement. Science 236 (4797), 48–54. 10.1126/science.236.4797.48 17759205

[B34] Grant T. N. Carola M. Zhang N. Finer J. J. (2017). Synthetic introns help identify sequences in the 5′ UTR intron of the Glycine max polyubiquitin (Gmubi) promoter that give increased promoter activity. Planta 245 (4), 849–860. 10.1007/s00425-016-2646-8 28070655

[B35] Grichko V. P. Glick B. R. Grishko V. I. Pauls K. P. (2005). Evaluation of tomato plants with constitutive, root-specific, and stress-induced ACC deaminase gene expression. Russian J. Plant Physiol. 52 (3), 359–364. 10.1007/s11183-005-0054-1

[B36] Guan X. Stege J. Kim M. Dahmani Z. Fan N. Heifetz P. (2002). Heritable endogenous gene regulation in plants with designed polydactyl zinc finger transcription factors. Proc. Natl. Acad. Sci. 99 (20), 13296–13301. 10.1073/pnas.192412899 12271125 PMC130627

[B37] Guilfoyle T. J. (1997). “The structure of plant gene promoters,” in Genetic engineering (Boston, MA: Springer), 15–47. 10.1007/978-1-4615-5925-2_2

[B38] Gupta M. DeKelver R. C. Palta A. Clifford C. Gopalan S. Miller J. C. (2012). Transcriptional activation of Brassica napus β-ketoacyl-ACP synthase II with an engineered zinc finger protein transcription factor. Plant Biotechnol. J. 10 (7), 783–791. 10.1111/j.1467-7652.2012.00695.x 22520333

[B39] Gurr S. J. Rushton P. J. (2005). Engineering plants with increased disease resistance: what are we going to express? Trends Biotechnol. 23 (6), 275–282. 10.1016/j.tibtech.2005.04.007 15922079

[B40] Gust A. A. Brunner F. Nürnberger T. (2010). Biotechnological concepts for improving plant innate immunity. Curr. Opin. Biotechnol. 21 (2), 204–210. 10.1016/j.copbio.2010.02.004 20181472

[B41] Hahne G. Horn M. Reski R. (2011). Plant biotechnology in support of the Millennium Goals. Plant Cell Rep. 30 (3), 245–247. 10.1007/s00299-010-0990-x 21279643

[B42] Higo K. Ugawa Y. Iwamoto M. Korenaga T. (1999). Plant cis-acting regulatory DNA elements (PLACE) database: 1999. Nucleic Acids Res. 27 (1), 297–300. 10.1093/nar/27.1.297 9847208 PMC148163

[B43] Hirai H. Tani T. Kikyo N. (2010). Structure and functions of powerful transactivators: VP16, MyoD and FoxA. Int. J. Dev. Biol. 54 (11-12), 1589. 10.1387/ijdb.103194hh 21404180 PMC3419751

[B44] Hiratsu K. Matsui K. Koyama T. Ohme-Takagi M. (2003). Dominant repression of target genes by chimeric repressors that include the EAR motif, a repression domain, in Arabidopsis. Plant J. 34 (5), 733–739. 10.1046/j.1365-313X.2003.01759.x 12787253

[B45] Hou L. Chen L. Wang J. Xu D. Dai L. Zhang H. (2012). Construction of stress responsive synthetic promoters and analysis of their activity in transgenic Arabidopsis thaliana. Plant Mol. Biol. Rep. 30 (6), 1496–1506. 10.1007/s11105-012-0464-0

[B46] Ishige F. Takaichi M. Foster R. Chua N. H. Oeda K. (1999). AG-box motif (GCCACGTGCC) tetramer confers high-level constitutive expression in dicot and monocot plants. Plant J. 18 (4), 443–448. 10.1046/j.1365-313X.1999.00456.x

[B47] Jiang X. L. He Z. M. Peng Z. Q. Qi Y. Chen Q. Yu S. Y. (2007). Cholera toxin B protein in transgenic tomato fruit induces systemic immune response in mice. Transgenic Res. 16 (2), 169–175. 10.1007/s11248-006-9023-5 17225072

[B48] Joshi C. P. (1987). An inspection of the domain between putative TATA box and translation start site in 79 plant genes. Nucleic Acids Res. 15 (16), 6643–6653. 10.1093/nar/15.16.6643 3628002 PMC306128

[B49] Kashima K. Yuki Y. Mejima M. Kurokawa S. Suzuki Y. Minakawa S. (2016). Good manufacturing practices production of a purification-free oral cholera vaccine expressed in transgenic rice plants. Plant Cell Rep. 35 (3), 667–679. 10.1007/s00299-015-1911-9 26661780

[B50] Kassaw T. K. Donayre-Torres A. J. Antunes M. S. Morey K. J. Medford J. I. (2018). Engineering synthetic regulatory circuits in plants. Plant Sci. 273, 13–22. 10.1016/j.plantsci.2018.04.005 29907304

[B51] Kim K. Y. Kwon S. Y. Lee H. S. Hur Y. Bang J. W. Kwak S. S. (2003). A novel oxidative stress-inducible peroxidase promoter from sweetpotato: molecular cloning and characterization in transgenic tobacco plants and cultured cells. Plant Mol. Bio 51 (6), 831–838. 10.1023/A:1023045218815 12777043

[B52] Kinkema M. Geijskes R. J. Shand K. Coleman H. D. De Lucca P. C. Palupe A. (2014). An improved chemically inducible gene switch that functions in the monocotyledonous plant sugar cane. Plant Mol. Biol. 84 (4–5), 443–454. 10.1007/s11103-013-0140-2 24142380

[B53] Koramutla M. K. Bhatt D. Negi M. Venkatachalam P. Jain P. K. Bhattacharya R. (2016). Strength, stability, and cis-motifs of in silico identified phloem-specific promoters in Brassica juncea (L.). Front. Plant Sci. 7, 457. 10.3389/fpls.2016.00457 27148290 PMC4834444

[B54] Koschmann J. Machens F. Becker M. Niemeyer J. Schulze J. Bülow L. (2012). Integration of bioinformatics and synthetic promoters leads to the discovery of novel elicitor-responsive cis-regulatory sequences in Arabidopsis. Plant Physiol. 160 (1), 178–191. 10.1104/pp.112.198259 22744985 PMC3440196

[B55] Koya V. Moayeri M. Leppla S. H. Daniell H. (2005). Plant-based vaccine: mice immunized with chloroplast-derived anthrax protective antigen survive anthrax lethal toxin challenge. Infect. Immun. 73 (12), 8266–8274. 10.1128/IAI.73.12.8266-8274.2005 16299323 PMC1307059

[B56] Kumar D. Patro S. Ghosh J. Das A. Maiti I. B. Dey N. (2012). Development of a salicylic acid inducible minimal sub-genomic transcript promoter from Figwort mosaic virus with enhanced root-and leaf-activity using TGACG motif rearrangement. Gene 503 (1), 36–47. 10.1016/j.gene.2012.04.053 22561698

[B57] Kumar D. Patro S. Ranjan R. Sahoo D. K. Maiti I. B. Dey N. (2011). Development of useful recombinant promoter and its expression analysis in different plant cells using confocal laser scanning microscopy. PloS One 6 (9), e24627. 10.1371/journal.pone.0024627 21931783 PMC3170401

[B58] Lam E. Chua N. H. (1991). Tetramer of a 21-base pair synthetic element confers seed expression and transcriptional enhancement in response to water stress and abscisic acid. J. Biol. Chem. 266 (26), 17131–17135. 1832669

[B59] Lescot M. Déhais P. Thijs G. Marchal K. Moreau Y. de Peer Y. (2002). PlantCARE, a database of plant cis-acting regulatory elements and a portal to tools for in silico analysis of promoter sequences. Nucleic Acids Res. 30 (1), 325–327. 10.1093/nar/30.1.325 11752327 PMC99092

[B60] Li M. Song B. Zhang Q. Liu X. Lin Y. Ou Y. (2013). A synthetic tuber-specific and cold-induced promoter is applicable in controlling potato cold-induced sweetening. Plant Physiol. Biochem. 67, 41–47. 10.1016/j.plaphy.2013.02.020 23542182

[B61] Liu S. Bao Y. (2009). Effects of copy number of an octopine synthase enhancer element and its distance from the TATA box on heterologous expression in transgenic tobacco. Acta Physiol. Plant. 31 (4), 705–710. 10.1007/s11738-009-0282-7

[B62] Liu W. Stewart C. N. (2016). Plant synthetic promoters and transcription factors. Curr. Opin. Biotechnol. 37, 36–44. 10.1016/j.copbio.2015.10.001 26524248

[B63] Liu W Mazarei M Peng Y Fethe MH Rudis MR Lin J Millwood RJ Arelli PR Stewart CN Jr (2014). Computational discovery of soybean promoter cis-regulatory elements for the construction of soybean cyst nematode-inducible synthetic promoters. Plant Biotechnol J. 12 (8), 1015–26. 10.1111/pbi.12206 24893752

[B64] Liu W. Mazarei M. Rudis M. R. Fethe M. H. Stewart C. N. (2011). Rapid *in vivo* analysis of synthetic promoters for plant pathogen phytosensing. BMC Biotechnol. 11 (1), 108. 10.1186/1472-6750-11-108 22093754 PMC3247077

[B65] Liu W. Yuan J. S. Stewart C. N. Jr. (2013). Advanced genetic tools for plant biotechnology. Nat. Rev. Genet. 14 (11), 781. 10.1038/nrg3583 24105275

[B66] Liu X. Li S. Yang W. Mu B. Jiao Y. Zhou X. (2018). Synthesis of seed-specific bidirectional promoters for metabolic engineering of anthocyanin-rich maize. Plant Cell Physiol. 59 (10), 1942–1955. 10.1093/pcp/pcy110 29917151

[B67] Madanala R. Gupta V. Pandey A. K. Srivastava S. Pandey V. Singh P. K. (2015). Tobacco chloroplasts as bioreactors for the production of recombinant superoxide dismutase in plants, an industrially useful enzyme. Plant Mol. Biol. Rep. 33 (4), 1107–1115. 10.1007/s11105-014-0805-2

[B68] Mahfouz M. M. Li L. Piatek M. Fang X. Mansour H. Bangarusamy D. K. (2012). Targeted transcriptional repression using a chimeric TALE-SRDX repressor protein. Plant Mol. Biol. 78 (3), 311–321. 10.1007/s11103-011-9866-x 22167390 PMC3259320

[B69] Mann D. G. King Z. R. Liu W. Joyce B. L. Percifield R. J. Hawkins J. S. (2011). Switchgrass (Panicum virgatum L.) polyubiquitin gene (PvUbi1 and PvUbi2) promoters for use in plant transformation. BMC Biotechnol. 11 (1), 74. 10.1186/1472-6750-11-74 21745390 PMC3161867

[B70] Matys V. Fricke E. Geffers R. Gößling E. Haubrock M. Hehl R. (2003). TRANSFAC^®^: transcriptional regulation, from patterns to profiles. Nucleic Acids Res. 31 (1), 374–378. 10.1093/nar/gkg108 12520026 PMC165555

[B71] Mehrotra R. Mehrotra S. (2010). Promoter activation by ACGT in response to salicylic and abscisic acids is differentially regulated by the spacing between two copies of the motif. J. Plant Physiol. 167 (14), 1214–1218. 10.1016/j.jplph.2010.04.005 20554077

[B72] Mehrotra R. Gupta G. Sethi R. Bhalothia P. Kumar N. Mehrotra S. (2011). Designer promoter: an artwork of cis engineering. Plant Mol. Biol. 75 (6), 527–536. 10.1007/s11103-011-9755-3 21327513

[B73] Mohan C. Jayanarayanan A. N. Narayanan S. (2017). Construction of a novel synthetic root-specific promoter and its characterization in transgenic tobacco plants. 3 Biotech. 7 (4), 234. 10.1007/s13205-017-0872-9 PMC5502212 28691155

[B74] Moose S. P. Mumm R. H. (2008). Molecular plant breeding as the foundation for 21st century crop improvement. Plant Physiol. 147 (3), 969–977. 10.1104/pp.108.118232 18612074 PMC2442525

[B75] Moradyar M. Motallebi M. Zamani M. R. Aghazadeh R. (2016). Pathogen-induced expression of chimeric chitinase gene containing synthetic promoter confers antifungal resistance in transgenic canola. In Vitro Cell. Dev. Biol.-Plant 52 (2), 119–129. 10.1007/s11627-016-9751-z

[B76] Na J. K. Metzger J. D. (2014). Chimeric promoter mediates guard cell-specific gene expression in tobacco under water deficit. Biotechnol. Lett. 36 (9), 1893–1899. 10.1007/s10529-014-1553-y 24863295

[B77] Nessler C. L. Lorence A. Chevone B. Mendes P., (2015). Stress tolerant transgenic plants over-expressing ascorbic acid and cell wall synthesis genes US9000-267B2, 160–185.

[B78] Nielsen A. A. Der B. S. Shin J. Vaidyanathan P. Paralanov V. Strychalski E. A. (2016). Genetic circuit design automation. Science 352 (6281), aac7341. 10.1126/science.aac7341 27034378

[B79] Novina C. D. Roy A. L. (1996). Core promoters and transcriptional control. Trends Genet. 12 (9), 351–355. 10.1016/S0168-9525(96)80017-5 8855664

[B80] Odell J. T. Nagy F. Chua N. H. (1985). Identification of DNA sequences required for activity of the cauliflower mosaic virus 35S promoter. Nature 313 (6005), 810. 10.1038/313810a0 3974711

[B81] Padidam M. (2003). Chemically regulated gene expression in plants. Curr. Opin. Plant Biol. 6 (2), 169–177. 10.1016/S1369-5266(03)00005-0 12667875

[B82] Park S. H. Yi N. Kim Y. S. Jeong M. H. Bang S. W. Choi Y. D. (2010). Analysis of five novel putative constitutive gene promoters in transgenic rice plants. J. Exp. Bot. 61 (9), 2459–2467. 10.1093/jxb/erq076 20363869 PMC2877896

[B83] Patro S. Maiti I. B. Dey N. (2013). Development of an efficient bi-directional promoter with tripartite enhancer employing three viral promoters. J. Biotechnol. 163 (3), 311–317. 10.1016/j.jbiotec.2012.11.009 23183382

[B84] Peramuna A. Bae H. Rasmussen E. K. Dueholm B. Waibel T. Critchley J. H. (2018). Evaluation of synthetic promoters in Physcomitrella patens. Biochem. Biophys. Res. Commun. 500 (2), 418–422. 10.1016/j.bbrc.2018.04.092 29660341

[B85] Peters B. Aidley J. Cadzow M. Twell D. Brownfield L., (2017). “Identification of cis-regulatory modules that function in the male germline of flowering plants,” in Plant Germline Development (New York, NY: Humana Press), 275–293. 10.1007/978-1-4939-7286-9_22 28936666

[B86] Petolino J. F. Davies J. P. (2013). Designed transcriptional regulators for trait development. Plant Sci. 201, 128–136. 10.1016/j.plantsci.2012.12.006 23352411

[B87] Porto M. S. Pinheiro M. P. N. Batista V. G. L. dos Santos R. C. de Albuquerque Melo Filho P. de Lima L. M. (2014). Plant promoters: an approach of structure and function. Mol. Biotechnol. 56 (1), 38–49. 10.1007/s12033-013-9713-1 24122284

[B88] Potenza C. Aleman L. Sengupta-Gopalan C. (2004). Targeting transgene expression in research, agricultural, and environmental applications: promoters used in plant transformation. In Vitro Cell. Dev. Biol.-Plant 40 (1), 1–22. 10.1079/IVP2003477

[B89] Ranjan R. Dey N. (2012). Development of vascular tissue and stress inducible hybrid–synthetic promoters through DOF-1 motifs rearrangement. Cell Biochem. Biophys. 63 (3), 235–245. 10.1007/s12013-012-9359-9 22610660

[B90] Römer P. Recht S. Lahaye T. (2009). A single plant resistance gene promoter engineered to recognize multiple TAL effectors from disparate pathogens. Proc. Natl. Acad. Sci. U. S. A. 106 (48), 20526–20531. 10.1073/pnas.0908812106 19910532 PMC2776607

[B91] Ruhlman T. Ahangari R. Devine A. Samsam M. Daniell H. (2007). Expression of cholera toxin B–proinsulin fusion protein in lettuce and tobacco chloroplasts–oral administration protects against development of insulitis in non-obese diabetic mice. Plant Biotechnol. J. 5 (4), 495–510. 10.1111/j.1467-7652.2007.00259.x 17490448 PMC2590789

[B92] Rushton P. J. Reinstädler A. Lipka V. Lippok B. Somssich I. E. (2002). Synthetic plant promoters containing defined regulatory elements provide novel insights into pathogen-and wound-induced signaling. Plant Cell 14 (4), 749–762. 10.1105/tpc.010412 11971132 PMC150679

[B93] Sahoo D. K. Sarkar S. Maiti I. B. Dey N. (2016). Novel Synthetic Promoters from the Cestrum Yellow Leaf Curling Virus. Methods Mol. Biol. 1482, 111–138. 10.1007/978-1-4939-6396-6_8 27557764

[B94] Saito K. Yamazaki M. Murakoshi I. (1992). Transgenic medicinal plants: Agrobacterium-mediated foreign gene transfer and production of secondary metabolites. J. Natural Prod. 55 (2), 149–162. 10.1021/np50080a001 1624938

[B95] Sakamoto A. Murata N. (2002). The role of glycine betaine in the protection of plants from stress: clues from transgenic plants. Plant Cell Environ. 25 (2), 163–171. 10.1046/j.0016-8025.2001.00790.x 11841661

[B96] Schöffl F. Rieping M. Baumann G. Bevan M. Angermüller S. (1989). The function of plant heat shock promoter elements in the regulated expression of chimaeric genes in transgenic tobacco. Mol. Gen. Genet. Mgg 217 (2–3), 246–253. 10.1007/BF02464888 2770695

[B97] Scranton M. A. Ostrand J. T. Georgianna D. R. Lofgren S. M. Li D. Ellis R. C. (2016). Synthetic promoters capable of driving robust nuclear gene expression in the green alga Chlamydomonas reinhardtii. Algal Res. 15, 135–142. 10.1016/j.algal.2016.02.011

[B98] Sharma A. K. Sharma M. K. (2009). Plants as bioreactors: Recent developments and emerging opportunities. Biotechnol. Adv. 27 (6), 811–832. 10.1016/j.biotechadv.2009.06.004 19576278 PMC7125752

[B99] Sharma H. C. Crouch J. H. Sharma K. K. Seetharama N. Hash C. T. (2002). Applications of biotechnology for crop improvement: prospects and constraints. Plant Sci. 163 (3), 381–395. 10.1016/S0168-9452(02)00133-4

[B100] Shehzadi A. Muhammad H. Abbas K. Ahmed Z. Saleem S. (2018). Effect plant disease resistance genes: recent applications and future perspectives. J. Innov. Bio.-Res. 1, 86–103.

[B101] Shepherd C. T. Scott M. P. (2009). Construction and evaluation of a maize (Zea mays) chimaeric promoter with activity in kernel endosperm and embryo. Biotechnol. Appl. Biochem. 52 (3), 233–243. 10.1042/BA20070269 18627354

[B102] Shokouhifar F. Zamani M. R. Motallebi M. Mousavi A. Malboobi M. A. (2011). Construction and functional analysis of pathogen-inducible synthetic promoters in Brassica napus. Biol. Plant. 55 (4), 689. 10.1007/s10535-011-0169-5

[B103] Shrestha A. Khan A. Dey N. (2018). Cis-Trans engineering: advances and perspectives on customized transcriptional regulation in plants. Mol. Plant. 11 (7), 886–898. 10.1016/j.molp.2018.05.008 29859265

[B104] Spitz F. Furlong E. E. (2012). Transcription factors: from enhancer binding to developmental control. Nat. Rev. Genet. 13 (9), 613. 10.1038/nrg3207 22868264

[B105] Srivastava R. Rai K. M. Srivastava M. Kumar V. Pandey B. Singh S. P. (2014). Distinct role of core promoter architecture in regulation of light-mediated responses in plant genes. Mol. Plant 7 (4), 626–641. 10.1093/mp/sst146 24177688

[B106] Sussex I. M. (2008). The scientific roots of modern plant biotechnology. Plant Cell 20 (5), 1189–1198. 10.1105/tpc.108.058735 18515500 PMC2438469

[B107] Timko M. P. Kausch A. P. Castresana C. Fassler J. Herrera-Estrella L. Van den Broeck G. (1985). Light regulation of plant gene expression by an upstream enhancer-like element. Nature 318 (6046), 579. 10.1038/318579a0 3865055

[B108] Tomlinson I. (2013). Doubling food production to feed the 9 billion: a critical perspective on a key discourse of food security in the UK. J. Rural Stud. 29, 81–90. 10.1016/j.jrurstud.2011.09.001

[B109] Tyagi A. K. (2001). Plant genes and their expression. Transgenic rice: a valuable monocot system for crop improvement and gene research. Curr. Sci.-BANGALORE- 80 (2), 161–169.

[B110] Tyagi A. K. Mohanty A. Bajaj S. Chaudhury A. Maheshwari S. C. (1999). Crit. Rev. Biotechnol. 19, 41–79. 10.1080/0738-859991229198

[B111] Varshney R. K. Bansal K. C. Aggarwal P. K. Datta S. K. Craufurd P. Q. (2011). Agricultural biotechnology for crop improvement in a variable climate: hope or hype? Trends Plant Sci. 16 (7), 363–371. 10.1016/j.tplants.2011.03.004 21497543

[B112] Vasil I. K. (2008). A history of plant biotechnology: from the cell theory of Schleiden and Schwann to biotech crops. Plant Cell Rep. 27 (9), 1423. 10.1007/s00299-008-0571-4 18612644

[B113] Venter M. (2007). Synthetic promoters: genetic control through cis engineering. Trends Plant Sci. 12 (3), 118–124. 10.1016/j.tplants.2007.01.002 17292658

[B114] Venter M. Botha F. C., (2010). “Synthetic promoter engineering,” in Plant Developmental Biology-Biotechnological Perspectives (Berlin, Heidelberg: Springer), 393–414. 10.1007/978-3-642-04670-4_20

[B115] Venter M. Zwiegelaar J. P., (2017). U.S. Patent No. 9,670,497. Washington, DC: U.S. Patent and Trademark Office.

[B116] Wang W. Vinocur B. Altman A. (2003). Plant responses to drought, salinity and extreme temperatures: towards genetic engineering for stress tolerance. Planta 218 (1), 1–14. 10.1007/s00425-003-1105-5 14513379

[B117] Wang R. Zhu M. Ye R. Liu Z. Zhou F. Chen H. (2015). Novel green tissue-specific synthetic promoters and cis-regulatory elements in rice. Sci. Rep. 5, 18256. 10.1038/srep18256 26655679 PMC4676006

[B118] Wever W. McCallum E. J. Chakravorty D. Cazzonelli C. I. Botella J. R. (2010). The 5′ untranslated region of the VR-ACS1 mRNA acts as a strong translational enhancer in plants. Transgenic Res. 19 (4), 667–674. 10.1007/s11248-009-9332-6 19816782

[B119] Xu L. Ye R. Zheng Y. Wang Z. Zhou P. Lin Y. (2010). Isolation of the endosperm-specific LPAAT gene promoter from coconut (Cocos nucifera L.) and its functional analysis in transgenic rice plants. Plant Cell Rep. 29 (9), 1061–1068. 10.1007/s00299-010-0892-y 20589378

[B120] Yamamoto Y. Y. Yoshioka Y. Hyakumachi M. Obokata J. (2011). Characteristics of core promoter types with respect to gene structure and expression in Arabidopsis thaliana. DNA Res. 18 (5), 333–342. 10.1093/dnares/dsr020 21745829 PMC3190954

[B121] Yang Z. Wang X. Xue J. Meng L. Li R. (2013). Identification and expression analysis of WRKY transcription factors in medicinal plant Catharanthus roseus. Sheng wu gong cheng xue bao. Chin. J. Biotechnol. 29 (6), 785–802. 24063238

[B122] Yeri S. B. Bhat R. S. Kuruvinashetti M. S. (2013). Functional analysis of synthetic promoters containing pathogen-responsive cis-elements. Mol. Plant Breed. 4, 270–276. 10.5376/mpb.2013.04.0034

[B123] Zhang C. Gai Y. Wang W. Zhu Y. Chen X. Jiang X. (2008). Construction and analysis of a plant transformation binary vector pBDGG harboring a bi-directional promoter fusing dual visible reporter genes. J. Genet. Genomics 35 (4), 245–249. 10.1016/S1673-8527(08)60034-X 18439982

[B124] Zhang W. McElroy D. Wu R. (1991). Analysis of rice Act1 5¢region activity in transgenic rice plants. Plant Cell 3 (11), 1155–1165. 10.1105/tpc.3.11.1155 1821763 PMC160082

[B125] Zhu Z. Gao J. Yang J. X. Wang X. Y. Ren G. D. Ding Y. L. (2015). Synthetic promoters consisting of defined cis-acting elements link multiple signaling pathways to probenazole-inducible system. J. Zhejiang Univ.-Sci. B 16 (4), 253–263. 10.1631/jzus.B1400203 25845359 PMC4399426

[B126] Zuo J. Chua N. H. (2000). Chemical-inducible systems for regulated expression of plant genes. Curr. Opin. Biotechnol. 11 (2), 146–151. 10.1016/S0958-1669(00)00073-2 10753773

